# Pipkin type III fractures: a narrative review of literature and comparative opinion on ORIF vs. arthroplasty

**DOI:** 10.3389/fsurg.2025.1554603

**Published:** 2025-08-18

**Authors:** Sujan Shakya, Qing Zhang, Yi Wen, Xinag Wen, Long Cheng

**Affiliations:** West China School of Medicine, West China Hospital, Sichuan University, Chengdu, China

**Keywords:** femoral head fracture, Pipkin III fracture, ORIF, THA, avascular necrosis

## Abstract

Pipkin Type III is extremely rare and is associated with worse prognosis and several complications. There is still no consensus on the management of injuries and whether these fractures should be treated surgically with ORIF or arthroplasty. Pipkin type III involves a combination of ipsilateral femoral head and neck fractures. Physioanatomical complexity of this fracture is often challenging, with a poorer prognosis than any other subgroups. The most common complications after management of Pipkin Type III are avascular necrosis of the femoral head and post-traumatic osteoarthritis. The treatment modality with open reduction and internal fixation (ORIF) or total hip arthroplasty (THA) varies according to the severity of the fracture, patient age, time to surgery, and intraoperative findings. Reoperation and surgical intervention pose significant economic burden, functional impairment, and quality of life. This comprehensive review on Pipkin type III demonstrates a throughout exploration of the existing research publications and case studies, focusing on current understanding treatment management strategies, outcome associated and decision-making frameworks with algorithms for decision schemes. By incorporating additional references of our institutional experiences, it aims to expand the current body of knowledge on Pipkin III fractures, offering fresh perspectives and crucial insights for surgeons in decision-making processes.

## Introduction

A Pipkin fracture, as described by Birkett in 1869, constitutes traumatic hip dislocation and femoral head fracture (1). These fractures and dislocations are often the result of high-energy trauma due to road traffic accidents (RTA), which account for approximately 5%–15% (2). Pipkin categorized injuries based on the location of the head fracture in relation to the fovea and associated lesions on the femoral neck or acetabulum (3). According to the classification system, subtype Pipkin III constitutes a combination of ipsilateral femoral head fractures and femoral neck fractures. They have the lowest percentile, representing only 8.6% of all Pipkin fractures (4). Based on the fact that the physio-anatomical complexity of the hip joint during reconstruction is often challenging with a poorer prognosis than the other sub-group. Regardless of the type of treatment, such as open reduction and internal fixation (ORIF) or total hip arthroplasty (THA), short- and long-term complications persist with vascular necrosis (AVN), post-traumatic osteoarthritis (PTA), stiffness, non-union, sciatic nerve palsy, and heterotopic ossification (HO), which lead to potentially varying degrees of disability in patients' outcomes. The aim of this extensive review of Pipkin type III fractures provides a comprehensive examination of current research publications and case reports, emphasizing the present understanding of treatment approaches, related outcomes, and decision-making protocols with algorithmic frameworks. By integrating additional references from our institution's experiences, this investigation sought to broaden the existing knowledge base on Pipkin III fractures, providing novel perspectives and essential insights to assist surgeons in their decision-making processes.

### Injury mechanism of Pipkin type III

Usually, Pipkin type III fractures occur when the hip is in greater than 60° of flexion, although the patient is not sure about the position of the limb in several studies (5). However, the mechanism of Pipkin type III femoral head fractures can be described as the application of two forces to the hip joint. The first axial force causes hip dislocation and femoral head fracture, and the second force after dislocation shears the femoral head against the iliac wing and causes a femoral neck fracture.

The initial trauma of the femoral head dislocation itself causes a loss of biomechanical stability of the hip joint. The anatomical components include the labrum, depth of the acetabulum, joint capsule, muscular supports, and surrounding ligaments (6). The majority of iliofemoral ligaments from the anterior aspect are much stronger, whereas the ischiofemoral ligament found posteriorly is vulnerable despite the dynamic gluteal muscles (7). Patients with posterior dislocation present in the emergency department with adducted, flexed, internally rotated, and shortened limbs.

Similarly, femoral neck fractures are common in young patients because of high-energy trauma and low-energy falls in elderly patients. Femoral neck fractures are considered intracapsular, and their healing potential is affected by the lack of periosteal surroundings that limit callus formation during rapid potential healing. According to Garden's classification, most high-energy accidents are vertical fractures (Pauwels type III) or Type III and IV femoral neck fractures. They are associated with compromised or disrupted blood supply, increased intracapsular pressure, and subsequently, decreased head perfusion. In our observation, in a Pipkin III fracture, the intact femur was completely disconnected from the head. Furthermore, dislocation significantly disrupted the vascular components of the medial femoral circumflex artery. Therefore, close reduction may not have been achieved. Furthermore, dislocation is associated with sciatic nerve injury, less commonly with peroneal branch and lumbosacral root injury due to direct compression by the femoral head or fractured fragments in the posterior wall.

### Literatures reviews on Pipkin type III and comparative treatment managements

After reviewing some of the largest series of published Pipkin fractures, the outcome of surgical management was distressing. Therefore, we conducted a literature review of all Pipkin III fractures, and the prognosis for those injuries is relatively poor, as shown in Table 1. The literature review includes all English-language publications from PubMed, Embase, and Google Scholar databases, covering original articles, case reports, and abstracts from 2000 to 2024 on Pipkin type III fractures. After thorough screening, only 21 publications were found relevant, specifically addressing Pipkin type III fractures, while other studies were excluded for lacking specific information on these fractures.

**Table 1:** The evidence of studies on the assessment of all Pipkin type III fractures with authors, publication date, demographic information, treatment plans, surgical approach, complications, and functional outcomes, which were included in this review.

Study/Year	Study design	Age/Sex	Cause	Time of operation (average hours-days)	Treatment plan	Surgical approach	Complications	Mean F/U	Functional outcome	Secondary operation
Stannard et al. UK (15)	Case series (1/22)	33/F	Traffic accident	1 day	ORIF-3 mm cannulated screws X3 with washers	Posterior (K–L)	AVN	24 months	Poor	—
Yamamoto et al. Japan (16)	Case series (1/10)	26/M	Traffic accident	3.2 days	Arthroscopic debridement	—	AVN within 1 year	7 years	Poor	—
Guimaraes et al. Brazil (17)	Case series (2/13)	32/M, 30/M	Traffic accident	—	THA	—	—	—	—	—
Tonetti et al. France (10)	Case series (4/10)	Mean age 37.1 years	Traffic accident	4.3 days	1 THA, 2-Fragment removal, 1-ORIF	Posterior approach (K–L)	AVN	3–6 months	Poor	—
Kokubo et al. Japan (12)	Case series (2/12)	80/F, 78/F	Traffic accident	5 h and 7 h	THA	Posterolateral	—	6 years 8 years	—	—
Park et al. Korea (18)	Case series (5/9)	44/M,36/M, 43/F,72/M, 57/F	Traffic accident	2.2 days	2-ORIF, 3-THA	Trochanteric Flip osteotomy	HO, AVN		Poor	—
Singaravadivelu et al. India (19)	Case report (1)	28/M	Traffic accident	—	2-THA	Posterior approach (K–L)	AVN	8 months	Poor	—
Scolaro et al. USA (11)	Case series (7/147)	Mean age 39.2 years	—	—	7-ORIF	Anterior (S-P) and posterior (Kocher–Langenbeck)	Fixation failures/AVN	12.4 weeks	Poor	2 pts–THA and 1-hemi-arthroplasty
Yu et al. China (14)	Case series (6/19)	Mean age 40.2 years	Traffic accident	48 h-7 days	1-THA, 2-cannulated screws (ORIF), 3-closed cannulated screws	Posterior (K–L) for ORIF and THA,	1-AVN	18 months	3 cases-good, 2 cases-Fair, 1 case—Poor	—
Zhao et al. China (20)	Case report (1)	34/M	Traffic accident	—	ORIF with cannulated screws and Herbert screws	Posterior (K–L)	—	1 year	Good	—
Keong et al. Singapore (9)	Case report (1)	35/F	Iatrogenic after reduction	—	ORIF with Herbert and cortical screws	Trochanteric Flip osteotomy	AVN	4 months	Poor	2^0^ THA in 4 months
Peng et al. Taiwan (21)	Case series (3/31)	Mean age 30 years	High energy trauma	2.9 days	ORIF cannulated screws	Anterior(S-P)/Posterior (Gibson Approach)	2-HO, 2-AVN	6 months-1 years	Poor	2-2^0^ THA. 1-f/u observation
Alyousif et al. Saudi Arabia (22)	Case report (1)	34/M	Iatrogenic after reduction	8 h	ORIF with cannulated screws and Herbert screws	Modified Gibson approach	—	26 months	Excellent	—
Mukhopadhaya et al. India (5)	Case report (1)	25/M	Motorbike accident	4 days	ORIF with multiple screws	Trochanteric Flip osteotomy	Mild AVN in 2 years	5 years	Excellent	—
Sen et al. India (23)	Case series (11/138)	Mean age 35.71 years	Mostly High energy trauma	—	5-ORIF, 6-THA	—	2-Infection, 2-AVN, 1-OA	3.57 years	4-Poor,1-Fair, 2-Good, 4-excellent	2-2^0^ THA, 2-Girdlestone arthroplasty
Enocson et al. Sweden (24)	Case series (1/47)	60/M	Falls	—	1-THA	—	—	3.5 years	—	—
Li et al. China (25)	Case report (1)	34/M	Iatrogenic after reduction	48 h	1-THA	—	—	—	—	—
YC Yoon et al. Korea (26)	Case series (4/34)	44/M, 31/M	Mostly Motor vehicle accidents	—	2-ORIF with cortical screws	Trochanteric Flip osteotomy	1-HO-2nd grade, AVN,	75 weeks 38 weeks	1-Poor, 2-Good	THA
Shakya et al. China (13)	Case series (8/50)	Median age 40 years	High energy trauma	6 h	5-ORIF, 3-THA	Anterior(S–P) + Lateral stab, Lateral stab, Posterior (K–L)	3-AVN, 3-PTA, 2-SNP	36 months	5-Fair 3-Poor	2 pts–2^0^ THA
Wang et al. China (27)	Case series (12)	Mean age 34.2	9-Traffic accidents, 2-falls, 1-Bruise injury	7.1 h	10-ORIF, 2-excision fragment	Posterior (K–L)	5-AVN, 1-HO, 1-Non-union	6 years	1-Excellent,4-Good,1-Fair, 6-Poor	6 pts–2^0^ THA
Paigude et al. India (28)	Case report (1)	35/M	Road Traffic Accident	24 h	ORIF with 2 headless screws, 3 cancellous cannulated screws	Posterior (K–L)	—	3 months	Good	—

THA, total hip arthroplasty; ORIF, open reduction and internal fixation; S–P, smith–petersen; K–L, kocher–langenbeck; AVN, avascular necrosis; HO, heterotopic ossifications; Pts, patients; 2^0^, THA-secondary THA.

Park et al. (8) retrospectively reviewed 65 femoral head fractures with hip dislocations. He observed the conversion of type III fractures from other types of Pipkin fractures in five out of nine cases in which close reduction had been attempted. They managed two of the five type III fractures using ORIF, which had an unsatisfactory outcome. THA was performed at 7 and 14 months after AVN. Therefore, the author suggested not attempting a close reduction in such injuries. A similar situation was also encountered by Keong et al. (9) in the case of iatrogenic Pipkin, Type III fracture after an attempt was made for hip relocation in 35 older women with posterior hip dislocation and femoral head fracture. He reported osteonecrosis of the femoral head four months after osteosynthesis with a headless compression screw of 4.5 mm and cortical screws of 6.5 mm which were ultimately converted to arthroplasty.

Tonetti et al. (10) published a retrospective series of 110 pipkin fractures. Out of which 4 Pipkin III fractures were treated. One patient underwent first-intention THA, while the remaining three underwent conversion to THR after ORIF. Similarly, Scolaro et al. (11) published a series of 147 pipkin fractures. Of these, seven were type III fractures that were initially managed with ORIF. Unfortunately, all of them failed operative fixation, leading to the conclusion that the Pipkin III fractures were catastrophic. As a result, all the affected patients underwent conversion to THR. In a case series, Kokubo et al. (12) reported on two elderly individuals with Pipkin Type III fractures. Both the patients were treated with THA. The authors suggested that open reduction and internal fixation should be the preferred treatment approaches for younger patients with Pipkin Type III fractures. However, they proposed that THA may be a suitable option for older patients with this type of injury.

A retrospective analysis of 50 individuals with Pipkin fractures was published by Shakya et al. (13) There were eight Pipkin type III patients in this study. Three (37%) patients underwent THA with the primary intention, while two (40%) of the five (63%) patients who underwent ORIF ultimately had to undergo secondary THA, which postulates that Pipkin III is predictive of THA.

Yu et al. (14) managed six Pipkin Type III patients using different surgical methods. One case of Type III was treated with Total Hip Arthroplasty, while the other two cases were treated with open reduction by cannulated screw fixation. The remaining three patients underwent closed reduction with cannulated screws. Despite the various functional outcomes among these patients, the authors stated that the rate of femoral head necrosis would increase enormously owing to the destruction of the blood supply to the femoral head by open reduction.

In a study conducted by Wang et al. (27), among 12 patients with Pipkin III, 6 (50%) underwent ORIF, 5 (42%) developed osteonecrosis, and 1 (8%) developed non-union. The study concluded that it was difficult to achieve satisfactory functional outcomes when treating Pipkin type III femoral head fractures using ORIF, and primary THA may be considered.

**Figure 1:**
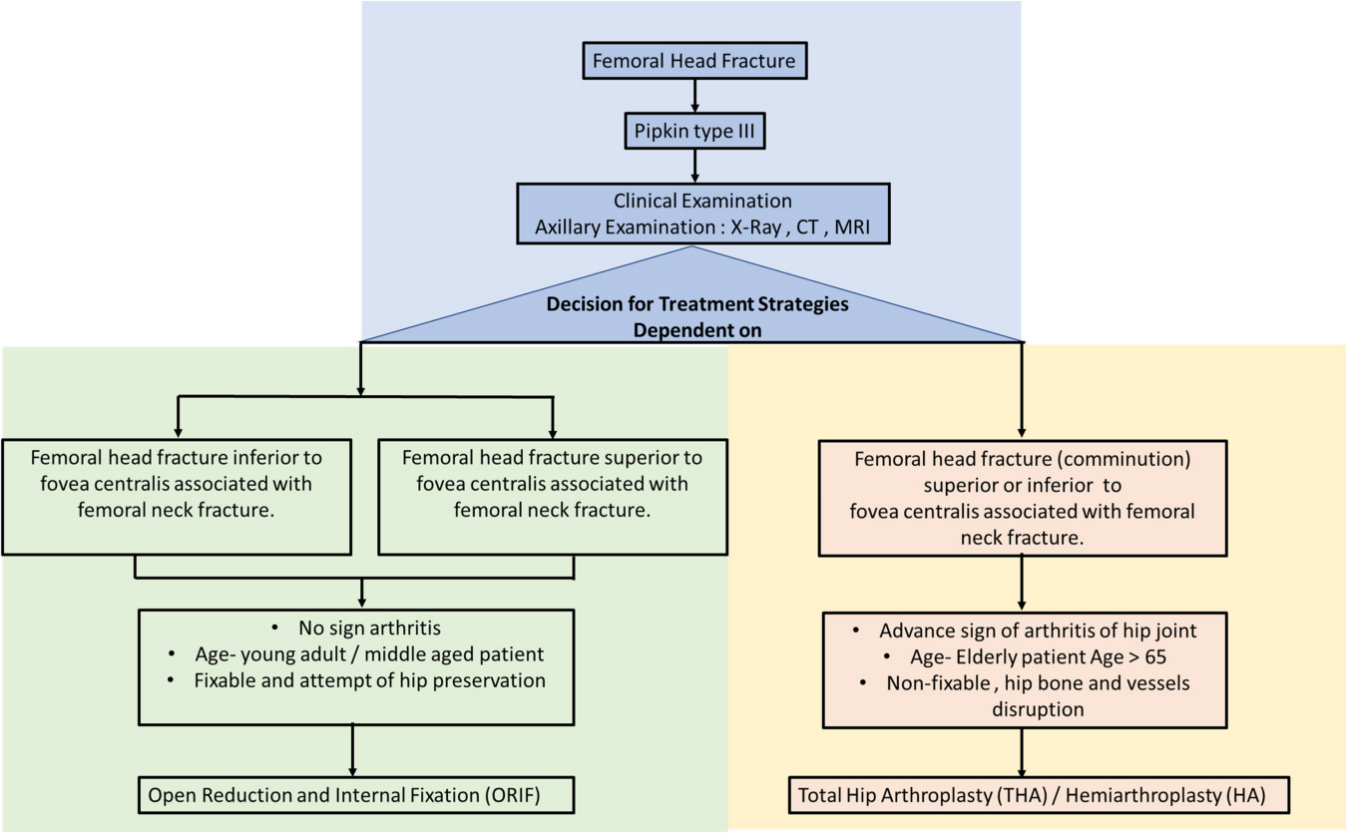
Flowchart illustrating recommended treatment algorithm for Pipkin type III femoral head fracture.

Based on the above larger series of studies, the authors suggested that arthroplasty should be strongly considered in cases of Pipkin Type III fractures. Therefore, we also proposed strategic treatment algorithm for Pipkin type III fractures for the decision making (Figure 1).

### Surgical methods on Pipkin type III

Despite advances in several surgical approaches for femoral head fractures, controversy and debate remain concerning effective surgical approaches. The surgical approaches used for the treatment of femoral head fractures are variable. These approaches include the Kocher–Langenbeck (posterior), Watson–Jones (anterolateral), Smith–Petersen (anterior), and Ludloff (medial) approaches (29).

Pipkin type III fractures were primarily treated using the posterior approach employing the Kocher–Langenbeck (K–L) or Trochanteric Flip techniques for open reduction and internal fixation (ORIF) (26, 27). Trochanteric osteotomy, Gibson approach, and a combination of anterior (Smith–Petersen) and lateral stab incisions for cannulated screw fixation have also been reported (13, 21, 22). In some cases, a combined anterior (Smith–Petersen) and posterior (Kocher–Langenbeck) approach has been used for fragment removal and fracture fixation. Compared to other surgical approaches, the posterior approach is associated with an increased incidence of osteonecrosis, as the majority of the blood supply to the hip comes from the posterior MFCA deep branch, which is endangered by the posterior approach (30). The incidence of HO was also higher with the posterior K–L approach.

Although the anterior approach causes less damage to the blood supply to the femoral head, the increased risk of HO and poor intraoperative visualization of the posterior structure of the hip and femoral neck are limited in Pipkin type III. In addition, it is difficult to reduce a femoral head fracture and femoral neck in a non-reducible Pipkin type III dislocation through an anterior approach.

**Figure 2:**
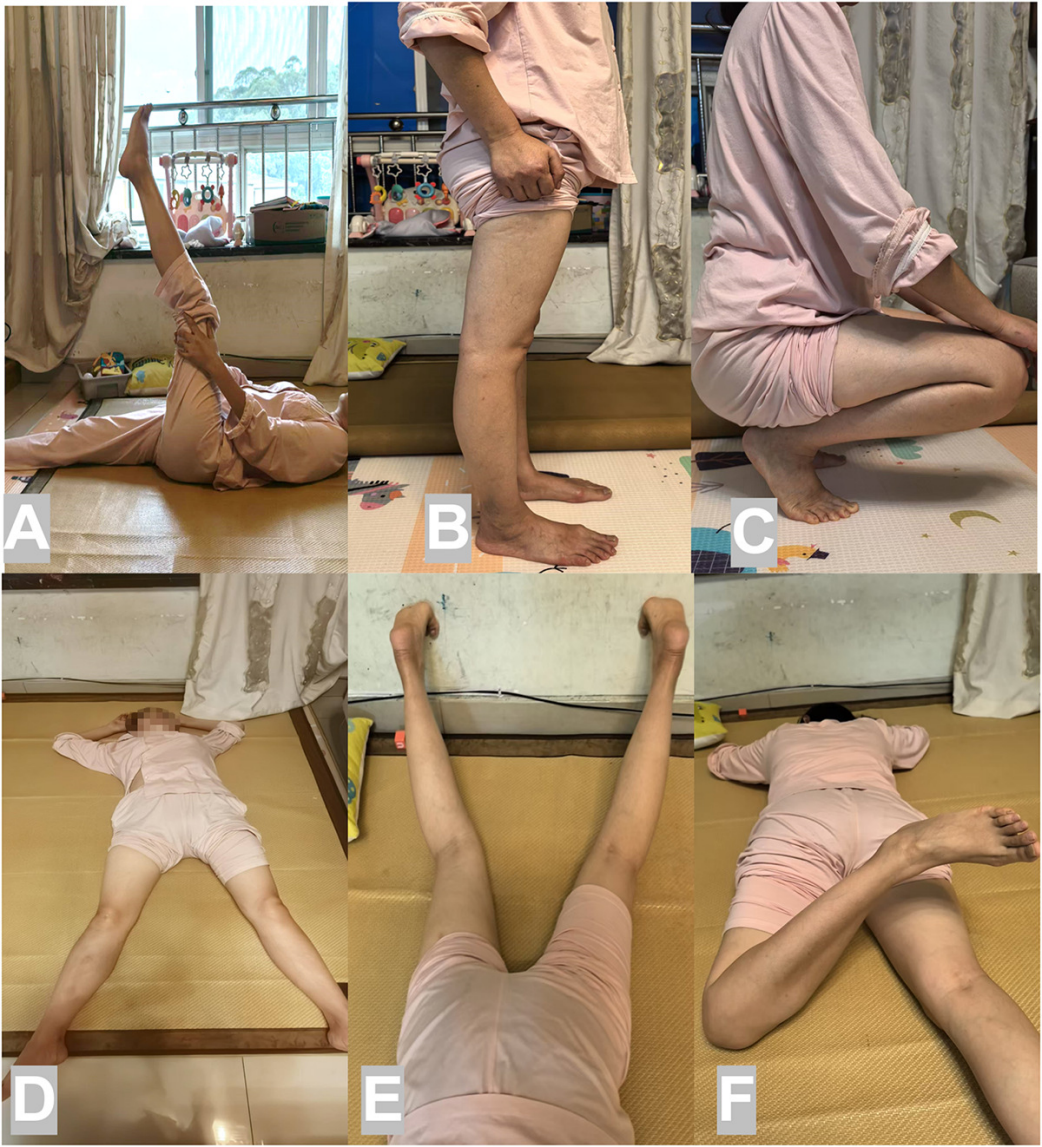
**(A)** Hip joint straight leg raising **(B)** Hip joint extension in standing position. **(C)** Complete squatting with flexion of hip joint. **(D)** Hip abduction in supine position. **(E)** Normal Internal rotation **(F)** Normal external rotation and abduction.

In our previous study, we described the concept and strategy of using the combined surgical window approach for Pipkin III. Here, reduction and fixation of the femoral head were achieved with Herbert screws using the anterior S–P approach. In the same window, reduction and preliminary fixation of the femoral neck were achieved, and a separate lateral stab approach was used for cannulated screw implant insertion (13). An x-ray conducted four years subsequently revealed the removal of the cannulated screws from the right hip, while the Herbert screws remained *in situ* on the femoral head. There were no indications of avascular necrosis or post-traumatic arthritis, and the hip exhibited a functionally adequate range of motion (Figure 2).

In addition to percutaneous femoral neck fixation strategies after a successful closed reduction of the femoral head fragment, a lateral or anterolateral approach may be an adequate option. An x-ray taken five years later displays the removal of the cannulated screws from the right hip, with the Herbert screws still in place on the femoral head with no indications of avascular necrosis or post-traumatic arthritis.

A notable surgical innovation was introduced by Ganz et al. in 2001, which enabled full visualization of the hip and femoral head (31). This technique, involving digastric flip osteotomy of the greater trochanter, was designed to address complex hip fractures, with a particular focus on Pipkin type III fractures. This procedure significantly improved the surgical management of these challenging cases. This method preserves the deep branch of the medial femoral circumflex artery and allows excellent visualization of the hip, including the femoral head and neck. Notably, the Ganz approach has been reported to have a lower rate of femoral head osteonecrosis.

The Ganz technique may be the preferred choice for treating Pipkin type III femoral head fractures when initial open reduction and internal fixation (ORIF) is selected. Nevertheless, this technique carries certain risks, including the possibility of non-union following trochanteric osteotomy. Additional potential complications include fracture non-union, dislocation of the osteotomized trochanter, and trochanteric bursa inflammation (32).

In 2018, Trikha et al. (33) demonstrated decreased complication rates and positive clinical outcomes in patients with complex acetabular or femoral head fractures treated with flip trochanter osteotomy. Lin et al. (34) corroborated these findings using an identical surgical technique. Their investigation, involving Pipkin I or II fractures, revealed that 77.3% achieved excellent or good results evaluated through MdA for clinical outcome and Thompson-Epstein for radiological outcome.

In a review conducted by Kloub et al. (35), odds ratio analysis demonstrated a significantly lower incidence of AVN in trochanteric-flip osteotomy than in anterior exposure (2.81 times, *p* = 0.008) and the classic Kocher–Langenbeck approach (2.19 times, *p* = 0.048). Moreover, flip osteotomy exhibited a 1.88 times lower occurrence of heterotopic ossification (all Brooker stages) than anterior exposure (*p* = 0.013).

Therefore, the Ganz flip approach (Gibson or K–L interval) is an emerging choice once initial ORIF has been chosen for treating Pipkin type III femoral head fractures.

In recent times, hip arthroscopy has emerged as a valuable technique increasingly employed for minimally invasive surgeries aimed at diagnosing and treating hip injuries. This procedure is frequently utilized to remove loose bodies or bone fragments within the joint and to clean up the labrum and ligamentum teres after damage to the posterior wall of the acetabulum or femoral head following hip reductions (36).

There has been a 93.8% success rate in patients with arthroscopically deployed loose body extractions, with good prognosis and few reported fatal complications (37). A systematic review and meta-analysis of Pipkin I fracture suggested that surgical excision has the best functional outcome, whereas fixation may have higher AVN and osteoarthritis. Thus, operating on type 1 fractures situated at lateral weight-bearing using arthroscopy-assisted fixation has advantages over open reduction (37–39). Likewise, Type 2 fractures in the infra-foveal area are more prone to be easily fixed in hip abduction, flexion, and external or internal rotation with better visualization during arthroscopy procedures (40). As a result, arthroscopy assist fixation of femoral head appears to be suitable primarily for addressing Pipkin type I and II fractures. With the current methods, tools, and expertise of skilled arthroscopists, hip arthroscopy is a complementary option for managing stable fracture of femoral head after the fixation of femoral neck in pipkin type III with appropriate management such as minimal invasive percutaneous fixation.

Furthermore, in selected patients, arthroscopy may enable intervention of both acetabular and femoral pathologies in the same session by closed means (41). Analysis by Chen et al. of hip scope–assisted surgery for Pipkin Type I and II femoral head fractures showed excellent and good results after fragment excision or fixation. No significant differences in operative time, VAS score, or hospital stay were found between groups. The excision group showed better outcomes than the fixation group according to mHHS (*P* = 0.009), similar to open surgery (40).

Following traditional surgical treatment for hip fracture-dislocations, major complications of AVN, osteoarthritis, and heterotopic ossification between 4% and 78% have been reported after the first 5 years of trauma (37). There are insufficient data to compare complication rates between ORIF and arthroscopy for hip fractures; however, arthroscopy may decrease complication rates, increase union rates, and yield excellent HHS results. It is important to note that osteoarthritis and avascular necrosis development are also associated with trauma severity, not just the treatment modality. Patients treated with arthroscopy assisted are mostly selected cases with less severe injuries (42).

Consequently, as supported by previous evidence-based studies, hip arthroscopy is an effective and less invasive method for addressing Pipkin Type I and II femoral head fractures, as well as Pipkin III after the femoral neck has been treated, resulting in good clinical outcomes.

## Discussion

Pipkin type III fractures result from high-energy hip trauma. A major concern is that it is the least frequent fracture with dual insult to the femoral head and neck, which is considered a catastrophic situation in orthopedic trauma. Upon preserving hip congruency, Pipkin type III fractures may not have favorable surgical outcomes as per the previously published literatures in the Table 1.

**Figure 3:**
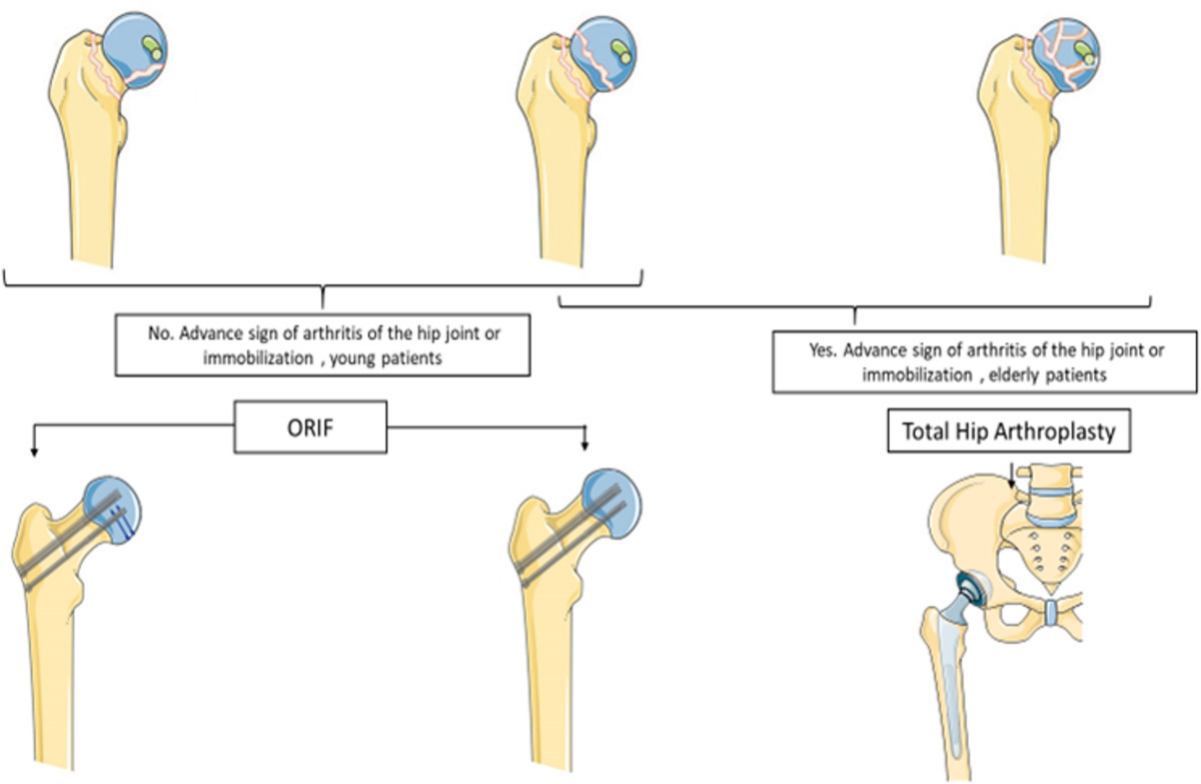
Illustrative diagram of recommended treatment variables for Pipkin type III fractures. Attempts for hip preservation in young patients with ORIF in the femoral head can be stabilized superiorly or inferiorly in fractured fragments with a countersunk or headless compression screw subchondral. The Smith-Peterson approach, which reduces surgical duration and blood loss, facilitates this stabilization. The stabilization of the femoral neck can be performed using percutaneous reduction with cannulated screws. Total hip replacement is selected for comminuted femoral head fractures with advanced arthritis or restricted joints in the elderly patients.

Despite head-preserving techniques, high-degree lesions in vertical neck fractures and fragments of the femoral head (anterior/inferior) with internal fixation, especially in young patients, can lead to osteonecrosis of the femoral head. Multiple cannulated screw fixation (CSF) and Herbert screws are widely accepted approaches for the management of such patients (Figure 3). The disability and consequences of requiring revision surgery or conversion to arthroplasty are 20%–36% (43). The failure rate due to AVN can be even higher for Pipkin III fractures.

**Figure 4:**
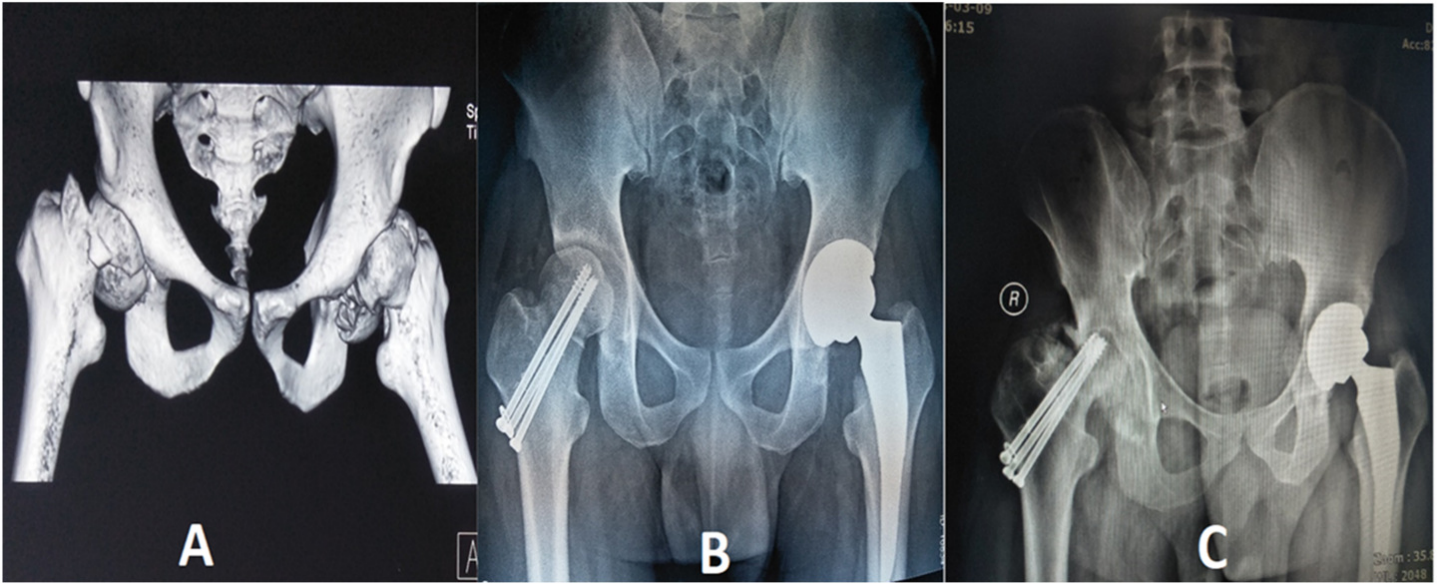
A 35-year-old man's **(A)** pre-operative 3D CT scan of the hip joint showed bilateral Pipkin III fracture-dislocation of the femoral head and neck. The right femoral head had two-part irregular fractures and a sub capital femoral neck fracture. The left hip had a comminuted femoral head fracture and subcapital neck fracture. **(B)** Postoperative radiographs revealing open reduction and internal fixation with three cannulated screws on the right hip and primary Total Hip Arthroplasty of the left hip. **(C)** Plain radiograph showing right-sided femoral head avascular necrosis with progressive shrinkage and collapse of the acetabulum dome within three years.

The development of osteonecrosis has been correlated with multiple factors including ischemic traumatic or non-traumatic intra-or extracapsular hip dislocation and fracture, age at the time of injury, degree of displacement, presence of posterior comminution, verticality of the fracture line, quality of reduction, and implant removal. Besides, corticosteroid therapy, chronic alcohol use, coagulopathy, and congenital causes frequently lead to AVN of the femoral head (44, 45). Moreover, prolonged dislocation of the joint and fracture accelerate the lack of blood supply to the femoral head, which may result in AVN of the femoral head. The remaining blood supply to the femoral head may be better preserved with an early and successful reduction. An attempt for prompt reduction with surgical management within six hours preferably decreases AVN (46). Osteonecrosis of the femoral head can occur anywhere between 6 months and many years after the initial injury; however, most cases present within 2 years (47). Therefore, patients should be followed-up for at least two years post-operatively to identify signs of osteonecrosis, both clinically and radiologically. The onset of AVN is insidious, and the signs and symptoms are minimal until they are in an advanced stage. Patients usually complain of localized pain in the groin radiating towards the anterior medial thigh or knee. Pain is exaggerated by weight-bearing activities, which are deep and throbbing, particularly at night. Supporting this evidence, Nam et al. explained the nature of asymptomatic osteonecrosis of the femoral head in which there were 105 asymptomatic. The study revealed that 43 AVN patients remained painless without collapse for 5 years or more (48). Osteosynthesis after Pipkin type III fractures can also cause mild pain and asymptomatic necrosis of the femoral head. Despite severe collapse of the weight-bearing portion, the patient continued his daily activities without much difficulty (Figure 4). In the case of implant collapse, the screw tip becomes prominent, colliding with the acetabulum dome, followed by the progressive loss of articular cartilage, leading to degeneration and osteoarthritis of the hip joint. Correspondingly, highly active patients have increased failure rates of ORIF and less favorable functional outcomes than elderly patients. However, assessing the stage of AVN with the use of MRI in Pipkin type III with post-ORIF stainless steel implantation is sometimes difficult due to metal artifacts (49).

Magnetic resonance imaging (MRI) is the imaging method of choice, with the highest sensitivity and specificity in comparison to plain radiography, computed tomography, or scintigraphy. It is the most useful screening tool for early diagnosis, quantitative evaluation of the extent of disease within the femoral head, and staging of the disease (50, 51). Ficat and Arlet are the most commonly used classifications for AVN (52). Taking into account the above evidence from our institutional experience, we believe that Pipkin III fractures are predictive of poorer outcomes with major complications, such as AVN. We advocate those adult patients with comminution of femoral head fractures and vertical fractures of the neck be managed with primary THA.

**Figure 5:**
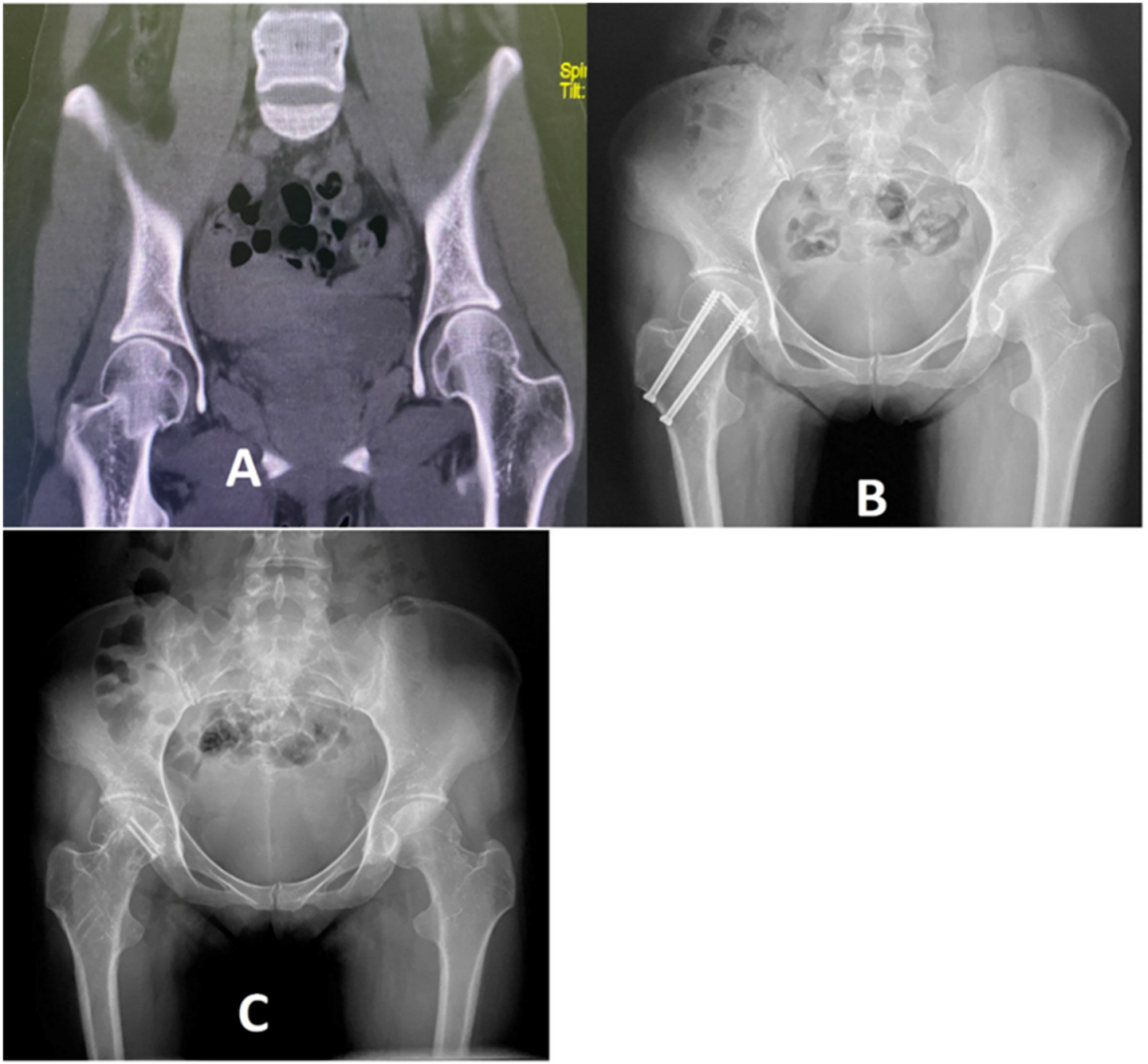
A 28y years old female who sustained a low energy fall at the workplace. **(A)** The pre-operative coronal view of CT scan revealed a right-sided femoral head and neck fracture. **(B)** The post-operative plain radiograph shows the internal fixation on the right femoral head and neck with two Herbert screws and three cannulated screws. **(C)** An x-ray taken four years later displays the removal of the cannulated screws from the right hip, with the Herbert screws still in place on the femoral head with no indications of avascular necrosis or post-traumatic arthritis.

Despite the above-mentioned strategy, ORIF should always be reserved for fractures with minimal breakage of the femoral head or non-displaced femoral neck fracture, with no evidence of dislocation, and young age (Figure 5). Despite strong arguments to consider arthroplasty for Pipkin III fractures, if anatomic reduction can be achieved, ORIF of Pipkin III fractures may still be reasonable, particularly in young patients. Additionally, low-energy iatrogenic femoral neck fractures may be easier to reduce anatomically and pose less risk of osteonecrosis; therefore, a single case may not predict the outcome for the more typical Pipkin III fracture treated with fixation (53). Additionally, safe surgical practice should be performed to protect the major deep medial circumflex artery branch and preserve the posterosuperior retinaculum. We also emphasized the early time to surgery in a safer manner when opting for ORIF. Otherwise, patients experience greater morbidity from injury due to collapse in ORIF and osteonecrosis.

Alternatively, hemiarthroplasty may also be an option for Pipkin type III fractures. However, erosion of the acetabular cartilage at some level is a complication that can result in pain and component migration, with the eventual need for revision Total Hip Arthroplasty (THA). The most common clinical etiology of acetabular erosion may be direct or indirect injury from initial trauma or wear and tear of the native cartilage. In contrast, non-anatomical artificial weight-bearing metals, polyethylene, or cement particles lead to an inflammatory reaction that can cause osteolysis and cartilage degeneration (54, 55). However, bipolar hemiarthroplasty with a metal-polyethylene interface has less stress and wear mechanisms in acetabular erosion. Ultimately, a long-term prognosis is necessary for THA (56).

It is well documented that the outcome of THA in the end-stage of hip disease, traumatic etiology of a femoral neck fracture, or Pipkin fractures is good with patient factors and implant characteristics (57, 58). THA has gained popularity as a solution for femoral neck fractures in elderly individuals with an active lifestyle, good general medical condition, and independent pre-injury mobilization status (59). THA in healthy individuals is associated with better patient-based outcomes but higher dislocation rates than hemiarthroplasty (60). Given the challenges of ORIF, salvage for THA following internal fixation of the femoral neck fracture has a significantly higher complication rate, such as infection, dislocation, and periprosthetic fracture, in comparison to primary THA (61) and without justifying doubt that holds the same for Pipkin III fractures. The mean time from primary osteosynthesis to additional THA in patients with Pipkin type III was approximately 27.7 months (27). Considering that the outcome of THA after ORIF surgery is suboptimal compared to the outcome of direct THA, most surgeons tend towards direct primary THA even at a young age because of the risk of AVN, keeping in mind that ORIF might eventually collapse when Pipkin type III is encountered (62).

THA implants typically last approximately 40 years, making them suitable for older individuals. However, the durability of these implants in younger and active patients can fluctuate according to their level of physical activity (63). These patients might need multiple revision surgeries throughout their lives because of implant deterioration or loosening, and even after THA, the implants may not fully accommodate their lifestyle needs. Consequently, it is crucial to consider various factors, including ORIF and primary THA, while also focusing on the surgical approach, long-term implant survival, post-operative care, and rehabilitation protocols (64). When selecting implants, it is important to evaluate weight-bearing surfaces, such as ceramic-on-ceramic and metal-on-polyethylene, as well as fixation methods involving cemented or cementless implants (65, 66).

**Figure 6:**
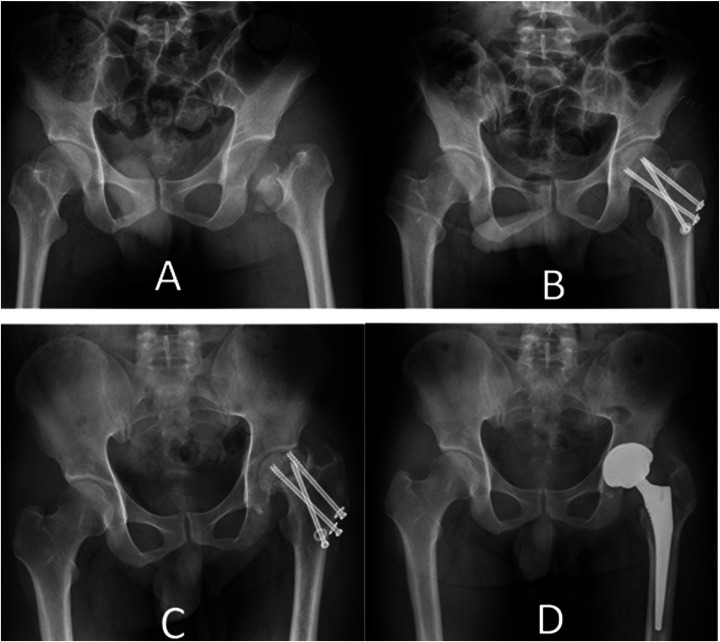
40-years old man sustained a high-speed vehicle accident **(A)** the pre-operative x-ray films revealed a pipkin III fracture involving the femoral head and neck on the left side. **(B)** The post-operative radiograph displayed internal fixation using three-cannulated screws. **(C)**Follow-up radiographs taken 18 months later indicated obvious implant failure and femoral head necrosis. **(D)** A postoperative radiograph taken two months after total hip arthroplasty is shown.

THA is the primary treatment for hip fractures in young individuals. Based on the literature, the 20-year prosthesis survivorship after primary THA in patients under 35 years of age is only 41%–66% (67–69). Therefore, it is prudent to choose an appropriate treatment option. Nevertheless, to achieve positive and excellent outcomes, both young and elderly patients undergoing THA have been continuously revolutionized with new and modern prosthetic component-designed implants and improved surgical techniques. New-generation Titanium Acetabular Component hip implants are ideal for extremely young and elderly patients undergoing THA to improve survivability and reduce complications (70). Thus, THA is preferable for Pipkin type III fractures (Figure 6).

The limitation of this literature review focuses specifically on Pipkin type III fractures and integrates our institution's experiences with existing research and case studies. Patients with other types of Pipkin fractures were excluded from the analysis. We posit that a substantial sample size would enhance the power of subgroup analyses based on factors such as age and timing of surgery or hip reduction. Various treatment methods, including ORIF, THA, and hemiarthroplasty, were collected for surgical management. However, a consistent comparative study focusing solely on ORIF and direct THA, with a long-term follow-up of at least 5–10 years, is necessary to assess the effectiveness of these two approaches for treating Pipkin III.

## Conclusion

In conclusion, the management of Pipkin type III fractures requires proper planning for either ORIF or THA. Our institutional experience concludes that surgical management with long-term strategies is essential to prevent complications such as AVN or post–traumatic arthritis, which leads to potentially varying degrees of disability in patient outcomes. Reoperation and surgical intervention pose significant economic burden, functional impairment, and quality of life. Therefore, identifying Pipkin Type III fractures, age group, severity, and expertise is essential for recommending surgical care. Owing to the lack of absolute recommendations and indications for management, the outcome is usually discouraging. In the past 20 years, the field of Pipkin type III has garnered increasing attention. This literature review can contribute additional insights and sources to the expanding body of knowledge on Pipkin III treatment. Consequently, it is crucial to conduct an extensive prospective investigation using validated outcome measures.
